# Predictors of developing *Mycobacterium kansasii* pulmonary disease within 1 year among patients with single isolation in multiple sputum samples: A retrospective, longitudinal, multicentre study

**DOI:** 10.1038/s41598-018-36255-w

**Published:** 2018-12-13

**Authors:** Hung-Ling Huang, Meng-Hsuan Cheng, Po-Liang Lu, Chia-Jung Liu, Inn-Wen Chong, Jann-Yuan Wang

**Affiliations:** 1 Division of Pulmonary and Critical Care Medicine, Department of Internal Medicine, Kaohsiung Medical University Hospital, Kaohsiung, Taiwan; 2 Graduate Institute of Medicine, College of Medicine, Kaohsiung Medical University, Kaohsiung, Taiwan; 3 Department of Laboratory Medicine, Kaohsiung Medical University Hospital, Kaohsiung, Taiwan; 4 Department of Internal Medicine, National Taiwan University Hospital, Taipei, Taiwan; 5 Departments of Respiratory Therapy, Kaohsiung Medical University Hospital, Kaohsiung, Taiwan

## Abstract

The clinical significance of a single *Mycobacterium kansasii* (MK) isolation in multiple sputum samples remains unknown. We conducted this study to evaluate the outcome and predictors of developing MK-pulmonary disease (PD) within 1 year among these patients. Patients with a single MK isolation from ≥3 sputum samples collected within 3 months and ≥2 follow-up sputum samples and chest radiography in the subsequent 9 months between 2008 and 2016 were included. The primary outcome was development of MK-PD within 1 year, with its predictors explored using multivariate logistic regression analysis. A total of 83 cases of a single MK isolation were identified. The mean age was 68.9 ± 17.9, with a male/female ratio of 1.96. Within 1 year, 16 (19%) cases progressed to MK-PD; risk factors included high acid-fast smear (AFS) grade (≥3), elementary occupation workers, and initial radiographic score >6, whereas coexistence with other nontuberculous mycobacterium species was protective. Among patients who developed MK-PD, all experienced radiographic progression, and 44% died within 1 year. Although a single MK isolation does not fulfil the diagnostic criteria of MK-PD, this disease may develop if having above-mentioned risk factors. Early anti-MK treatment should be considered for high-risk patients.

## Introduction

The burden of nontuberculous mycobacteria (NTM) pulmonary disease (PD) has been increasing worldwide in recent decades^1–3^. *Mycobacterium kansasii* (MK) is one of the most virulent species among all NTM species^4^, with similar clinical manifestation and radiographic characteristics of pulmonary tuberculosis (TB)^5,6^. Current reports reveal that both the number of respiratory isolates of MK and the incidence of MK-PD have increased worldwide^5–8^, including Taiwan^9^. The predisposing factors of MK-PD include structural lung disease and immunocompromised status of the host^7,10,11^. Water pollution exposure and occupational history have also been reported to be associated with the development of MK-PD^5,12^.

The presence of NTM in respiratory specimens does not indicate true infection, and the diagnosis of NTM-PD depends on composite microbiological and clinical criteria^1,13^; at least two positive sputum cultures of the same NTM species is required to fulfil the microbiological criteria from current guideline^1^. Therefore, a single NTM isolation from multiple respiratory specimens is usually considered colonization or contamination rather than indicative of a true pathogen. In a study conducted in South Korea, 26 (14%) of 190 patients with a single sputum isolation of pathogenic NTM met the diagnostic criteria for NTM-PD within a median follow-up period of 16 months, and none of the 8 patients with a single MK isolation had a subsequent positive culture^14^. Under this clinical entity, follow-up sputum sampling and clinical monitoring are necessary to diagnose NTM-PD; yet, the clinical outcome and optimal duration of follow-up remain uncertain^14,15^. Furthermore, patients may have different NTM species in the respiratory tract presenting as transitional, alternating, or simultaneous pattern in chronological order^1,16,17^. Little is known of the clinical significance of coexistence of different NTM species.

In Taiwan, MK is the third most common NTM causing PD, and the numbers of MK isolates increased 4.7-fold from 2010 to 2014^9^. Being familiar with the outcome of different clinical entities of MK is crucial in clinical practice. We therefore conducted this retrospective, longitudinal cohort study to investigate the predictors of developing MK-PD within 1 year among patients with single MK isolation from multiple sputum samples, with a special emphasis on coexistence of NTM species other than MK.

## Results

### Study population

**Figure 1:**
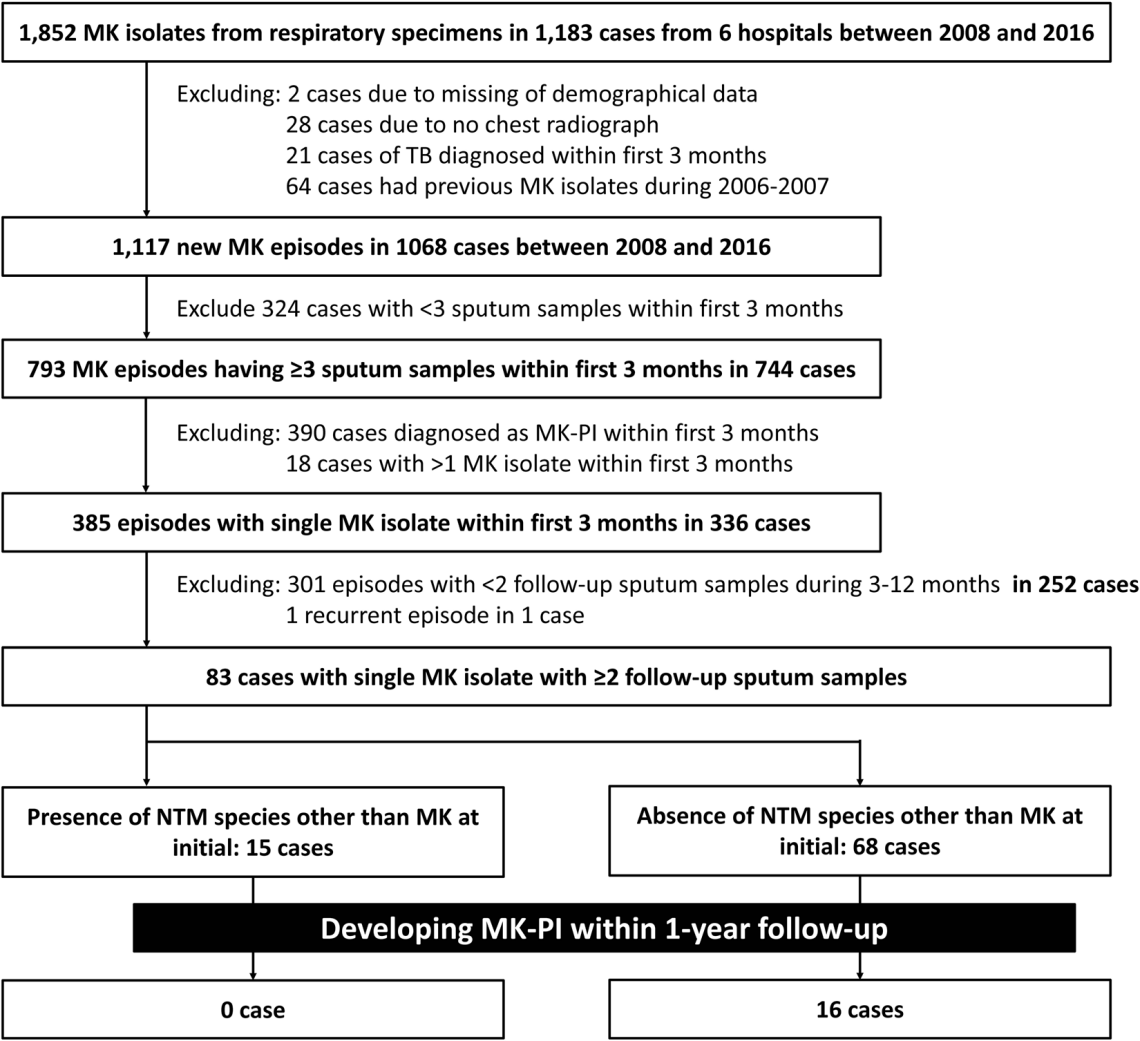
Flowchart of selection of new cases of *Mycobacterium kansasii* (MK) pulmonary disease (PD) in six hospitals.

Figure 1 shows the flowchart of patient selection and the enrolment criteria. Between 2008 and 2016, a total of 1,852 respiratory MK isolates in 1,183 patients were identified from six hospitals. By applying the selection criteria, a total of 83 (7.0%) cases of single MK isolation were finally selected for further analysis.

Among the 83 patients, the mean age of the patients was 68.9 ± 17.9 years, with a male/female ratio of 1.96. Among the 83 patients, 52% were ex-smokers and 54% had received education for <6 years. 20 (24%) cases were employed in elementary occupations. Of them, 18 were in industrialized areas, including 14 construction laborers and 4 iron-steel manufacturing laborers. Of the remaining two patients, one was a truck driver and the other was a cleaner. The most common pulmonary comorbidity was chronic obstructive pulmonary disease (COPD: 35%), and the most common systemic comorbidity was chronic kidney disease stages 3–5 (23%). Higher (but nonsignificantly) prevalence rates of COPD and pneumoconiosis (respectively, 50% vs. 28%, *p* = 0.086; 11% vs. 0%, *p* = 0.060) were noted in patients with elementary occupations from industrialized areas than in others. Fifteen (18%) patients had coexistence of NTM species other than MK, including *Mycobacterium avium* complex (MAC) in nine, *M. fortuitum* in two, one each for *M. abscessus* and *M. scrofulaceum*, and unidentified NTM species in two.

There was no significant difference in clinical characteristics between the 15 patients with coexistence of other NTM species and the other 68 without such coexistence (Table 1), except that the coexistence group had a significantly higher prevalence of congestive heart failure (33% vs. 7%, *p* = 0.018). The initial symptoms, laboratory data, lung function, radiographic findings, and sputum mycobacteriologic results were similar between the two groups.

**Table 1:** Clinical characteristics of patients with a single *Mycobacterium kansasii* isolation, stratified by coexistence of other nontuberculous mycobacteria species.

	Coexistence of other NTM (N = 15)	No coexistence of other NTM (N = 68)	*p*-value
Age (year)	69.5 ± 17.2	68.7 ± 18.2	0.877
Male sex	12 (80%)	43 (63%)	0.214
Body-mass index (kg/m^2^)	20.2 ± 4.2	19.8 ± 4.2	0.586
<18.5	5 (33%)	30 (44%)	0.476
Smoking status			
Never smoker	6 (40%)	25 (37%)	0.815
Ex-smoker	8 (53%)	35 (52%)	0.896
Current smoker	1 (7%)	8 (12%)	0.908
Alcoholism	5 (33%)	26 (38%)	0.722
Education period (n = 13, 65)			
<6 years	7 (54%)	35 (53%)	>0.999
6 ~ 9 years	2 (15%)	7 (11%)	0.582
9 ~ 12 years	1 (8%)	14 (22%)	0.271
≥12 years	3 (23%)	9 (14%)	0.405
Occupation history^a^			
Elementary occupations	1 (7%)	19 (28%)	0.103
Professionals	1 (7%)	1 (2%)	0.331
Technicians and associate professionals	0	1 (2%)	>0.999
Service and sales workers	3 (20%)	12 (17%)	0.830
Craft and related trades workers	0	3 (4%)	>0.999
Plant/machine operators and assemblers	1 (7%)	2 (3%)	0.455
Retired or unemployed	9 (60%)	29 (43%)	0.222
Pulmonary comorbidity			
Chronic obstructive pulmonary disease	4 (27%)	24 (35%)	0.735
Bronchiectasis	4 (27%)	17 (25%)	0.893
History of pulmonary tuberculosis	5 (33%)	18 (27%)	0.591
Lung cancer	2 (13%)	7 (10%)	0.663
Other pulmonary diseases	1 (7%)^b^	9 (13%)^c^	0.665
Systemic comorbidity			
Chronic kidney disease, stage 3–5	3 (20%)	16 (24%)	0.768
Congestive heart failure	5 (33%)	5 (7%)	0.018
Diabetes mellitus	7 (47%)	20 (29%)	0.197
Extra-pulmonary cancer	1 (7%)^d^	13 (19%)^e^	0.468
Steroid user^*f*^	0	8 (12%)	0.361
HIV infection	0	1 (2%)	>0.999
Other systemic diseases	0	6 (9%)^g^	0.563
Initial symptoms			
Sputum	10 (67%)	58 (85%)	0.090
Cough	11 (73%)	42 (62%)	0.399
Hemoptysis	1 (7%)	15 (22%)	0.281
Dyspnea	5 (33%)	21 (31%)	0.853
Initial laboratory data			
Leukocyte > 9000/uL	2 (13%)	24 (36%)	0.126
Segment > 70% (n = 13, 50)	3 (23%)	25 (50%)	0.119
Hemoglobin < 12 g/dL	9 (60%)	30 (46%)	0.309
Platelet count < 140 K/uL	2 (13%)	11 (17%)	0.751
C-reactive protein > 10 mg/L (n = 5, 40)	3 (60%)	27 (68%)	0.737
Aspartate transaminase > 40 U/L (n = 13,66)	1 (7%)	12 (19%)	0.444
Alanine transaminase > 40 U/L (n = 14, 66)	2 (13%)	12 (19%)	0.638
Creatinine > 1.4 mg/dL (n = 15, 67)	2 (13%)	13 (19%)	0.726
Albumin < 3.5 g/dL (n = 3, 20)	2 (67%)	14 (70%)	>0.999
Lung function (n = 8, 32)			
FEV_1_ (% of predicted)	74.2 ± 37.0	71.7 ± 36.9	0.584
FEV_1_/FVC	72.9 ± 10.0	72.2 ± 10.8	0.871
Obstructive type	3 (38%)	14 (44%)	0.959
Restrictive type	1 (13%)	8 (25%)	0.571
Radiographic finding			
Predominant pattern			
Fibrocavitory	1 (7%)	11 (16%)	0.360
Nodular bronchiectasis	0	9 (13%)	0.279
Multifocal involvement	12 (80%)	56 (81%)	>0.999
Initial radiographic score	5.5 ± 2.2	5.7 ± 3.3	0.818
Follow-up radiographic score	6.1 ± 3.6	6.8 ± 4.3	0.552
Initial sputum study			
Number of sputum samples	4.1 ± 1.3	3.8 ± 1.4	0.552
Acid-fast smear			
Negative	9 (60%)	53 (78%)	0.148
Low-grade positive (Gr. 1, 2)	6 (40%)	12 (18%)	0.065
High-grade positive (Gr. 3, 4)	0 (0%)	3 (4%)	>0.999

Abbreviations: FEV_1_: forced expiratory volume in first second; FVC: forced vital capacity; NTM: nontuberculous mycobacteria.

Data are number (percentage) or mean ± standard deviation.

^a^The occupational category was noted according to the International Standard Classification of Occupations, 2008 (ISCO-08)^28^.

^b^One had asthma.

^c^Three had interstitial lung disease, three had asthma, two had pneumoconiosis, and the remaining one had both interstitial lung disease and asthma.

^d^One had oesophageal cancer.

^e^Five had leukaemia and eight had solid organ tumours, including thymic cancer in two; prostate cancer in two; and one each for gastric cancer, papilla of Vater cancer, colon cancer, and basal cell carcinoma.

^f^Steroid user was defined as receiving at least 1 week with a dose of ≥30 mg/day of oral prednisone (or equivalent).

^g^Four had autoimmune disease, and two had liver cirrhosis.

### Outcome and risk factors of MK-PD

**Figure 2:**
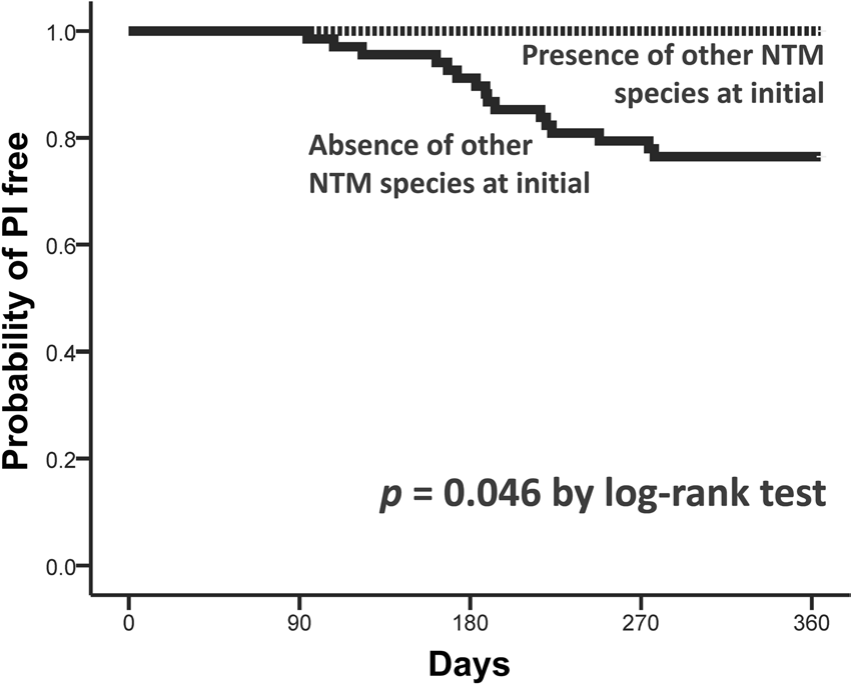
Kaplan–Meier curves for time to development of *Mycobacterium kansasii* (MK) pulmonary disease (PD), stratified by coexistence of nontuberculous mycobacteria (NTM) species other than MK.

Within 1 year, MK-PD developed in 16 (19%) patients, including 6 (38%) in the second quarter and 8 (50%) in the third quarter (Fig. 2). All of these patients experienced radiographic progression during follow-up. The radiographic findings during MK-PD development were FC pattern in 10 (63%) patients and NB pattern in 6 (38%), and all showed multifocal involvement.

Univariate logistical regression (Table 2, left panel) revealed that MK-PD was more likely to develop in patients with BMI < 18.5 kg/m^2^ (31% vs. 11%, *p* = 0.033), elementary occupations (60% vs. 6%, *p* < 0.001), COPD (56% vs. 12%, *p* = 0.010), steroid use (50% vs. 16%, *p* = 0.032), haemoptysis (38% vs. 15%, *p* = 0.047), leucocytosis (white blood cells >9000/μL; 42% vs. 9%, *p* = 0.001), haemoglobin <12 g/dL (28% vs. 12%, *p* = 0.034), high-grade (grade 3 or 4) positivity of sputum AFS (100% vs. 16%, *p* = 0.007), and initial radiographic score >6 (33% vs. 9%, *p* = 0.008). By contrast, coexistence with NTM species other than MK was associated with a lower risk of developing MK-PD (0% vs. 25%, *p* = 0.037).

**Table 2:** Univariate and multivariate logistic regression analysis for predictors of progression to *Mycobacterium kansasii* pulmonary disease.

Variables	Univariate (n = 83)		Multivariate (n = 65)^a^	
	OR (95% CI)	*p* value	OR (95% CI)	*p* value
Age > 65	2.27 (0.59–8.76)	0.236		
Male sex	0.72 (0.26–2.53)	0.723		
Body-mass index < 18.5	3.58 (1.11–11.55)	0.033	1.65 (0.05–7.54)	0.696
Elementary occupations^b^	22.12 (5.73–85.45)	<0.001	10.77 (1.65–70.51)	0.013
Chronic obstructive pulmonary disease	4.54 (1.44–14.29)	0.010	2.48 (0.24–25.74)	0.447
History of pulmonary tuberculosis	0.50 (0.13–1.95)	0.314		
Bronchiectasis	0.63 (0.16–2.46)	0.505		
Diabetes mellitus	0.24 (0.05–1.15)	0.073	0.27 (0.03–2.91)	0.280
Congestive heart failure	1.05 (0.20–5.52)	0.951		
Steroid use	5.25 (1.15–23.94)	0.032	3.58 (0.20–63.18)	0.383
Hemoptysis	3.42 (1.02–11.53)	0.047	1.67 (0.13–22.26)	0.698
Cough	2.93 (0.76–11.25)	0.118		
Sputum	1.69 (0.34–8.35)	0.523		
Dyspnea	1.97 (0.64–6.03)	0.238		
Leukocyte > 9000/uL	6.36 (2.02–20.05)	0.001	1.94 (0.27–13.87)	0.509
Hemoglobin < 12 g/dL	3.34 (1.06–10.57)	0.034	3.55 (0.48–26.09)	0.213
Initial radiographic scores > 6	5.38 (1.56–18.52)	0.008	10.25 (1.24–84.59)	0.031
Anti-MK treatment: intent to treat^c^	2.69 (0.76–9.44)	0.114		
Anti-MK treatment: per-protocol^d^	4.33 (0.56–33.29)	0.129		
High-grade (Gr. 3 or 4) positive for AFS^a^	32.59 (1.60–665.77)	0.007		
Co-existence of NTM species other than MK^a^	0.78 (0.68–0.88)	0.037		

Abbreviation: AFS, acid-fast smear; NTM, nontuberculous mycobacteria.

^a^Among the 15 episodes with coexistence of other NTM species, none progressed to MK-PD during 1-year follow-up, whereas 16 (24%) of the 68 without concomitant NTM species did (*p* = 0.037). Of the 68 episodes, 3 had high-grade positivity (grades 3 and 4) in sputum AFS, and all developed MK-PD, whereas 13 (20%) of the 65 episodes without high AFS grade did (*p* = 0.001). Thus, we conclude that coexistence with other NTM was a significant protector against MK-PD and that high AFS grade was a significant predictor for MK-PD. We excluded 18 episodes from the data in fitting the multivariate logistic model.

^b^The category of occupation was noted according to the International Standard Classification of Occupations, 2008 (ISCO-08)^28^.

^c^Patients who had ever received any drugs against MK, regardless of duration.

^d^Patients who had ever received combination chemotherapy against MK for more than 2 months in the first 3 months.

Multivariate logistic regression analysis was performed to investigate the independent predictors for developing MK-PD. We found a statistical separation phenomenon existed in two variables, coexistence with other NTM species and high-grade positivity in sputum AFS. Among the 15 episodes with coexistence of other NTM species, none progressed to MK-PD within the 1-year follow-up, whereas 16 (24%) of the 68 without concomitant other NTM species did (*p* = 0.037). Of the remaining 68 episodes, 3 had high-grade positivity (grades 3 and 4) in sputum AFS, with all developing into MK-PD; by contrast, 13 (20%) of the other 65 episodes without high AFS grade developed into MK-PD (*p* = 0.001). Therefore, we excluded above 18 episodes from the data in fitting the multivariate logistic model. Subsequent analysis of the remaining 65 episodes revealed that independent predictors of developing MK-PD within 1 year were an elementary occupation (odds ratio [95% confidence interval] = 10.77 [1.65–70.51], *p* = 0.013) and initial radiographic score >6 (10.25 [1.24–84.59], *p* = 0.031) (Table 2, right panel).

### One-year outcome

Treatment courses and 1-year mortality are summarized in Table 3. Treatment for MK was more frequently, though not significantly, prescribed in the 16 cases developing into MK-PD according to either intention-to-treat analysis (29% vs. 13%, *p* = 0.087) or per-protocol analysis (13% vs. 3%, *p* = 0.166). Of the 16 patients who developed MK-PD within 1 year, 7 (44%) died, 4 of whom died of MK-PD. By contrast, 6 (9%) of the 67 without development of MK-PD died (*p* = 0.002). Neither of the two patients with MK-PD who received standard anti-MK treatment died, whereas two patients who received transient anti-MK treatment died.

**Table 3:** Treatment and 1-year outcome of patients, stratified by progression to *Mycobacterium kansasii* pulmonary disease (MK-PD).

	Progressed to MK-PD (N = 16)	Not progress to MK-PD (N = 67)	*p*-value
**Treatment against MK**			
Intent to treat analysis^a^	5 (29%)	9 (13%)	0.087
Per-protocol analysis^b^	2 (13%)	2 (3%)	0.166
Mortality in one year	7 (44%)	6 (9%)	0.002
Time to mortality	245 (192–299)	286 (190–302)	0.681^*c*^
**Cause of Death**			
Sepsis with bacterial pathogen	3 (19%)	4 (6%)	
*Mycobacterium kansasii*	4 (25%)	0 (0%)	
Others	0	2 (3%)^*d*^	

Data are number (percentage) or median ± *interquartile range*.

*p* value was calculated using the chi-squared test unless otherwise mentioned. ^a^Patients who had ever received any drugs against MK, regardless of duration.

^b^Patients who had ever received combination chemotherapy against MK for more than 2 months in the first 3 months.

^c^*p* value was calculated by log-rank test.

^d^The cause of death was lung cancer in one and acute myocardial infarction in the other.

## Discussion

To our knowledge, this is the first longitudinal, multicentre study investigating the incidence and predictors of developing MK-PD within 1 year among patients with single MK isolation from multiple sputum samples. There were two major findings in this study. First, MK-PD developed in 19% of cases, with 88% of the MK-PD occurring in the second and third quarters after the index date. All patients who progressed to MK-PD had typical radiographic patterns with multifocal involvement, and radiographic progression occurred thereafter. The 1-year mortality rate was 44% in MK-PD patients with MK-PD, which was 4.9 times higher than in those without MK-PD. Second, high-grade sputum AFS at initial presentation, elementary occupations, and higher initial radiographic scores (>6) were independent risk factors for developing MK-PD within 1 year, whereas coexistence with other NTM species was protective.

Though a single respiratory isolation of pathogenic NTM, such as MAC, *M. abscessus*, and MK, is insufficient to establish the diagnosis of NTM-PD^1^, the results of previous studies suggest that it is clinically significant in appropriate clinical settings, especially in cavitary lung disease^5,17–19^. Some experts also suggest a lower diagnostic threshold for patients with positive respiratory cultures of MK, particularly in people living with human immunodeficiency virus^18,20,21^. Another study obtained the opposite result, showing that 14% of patients with a single sputum isolation of NTM eventually developed NTM-PD within a median follow-up period of 16 months^14^. Another retrospective observational study had a similar finding, showing that none of 18 patients with a single MK isolation developed MK-PD within a median of 12 months^22^. However, it’s difficult to draw a definite conclusion due to the limitation of power (small sample size). Therefore, an appropriate follow-up period is proposed to be essential to determine the clinical relevance of a single MK respiratory isolation^14,15^. The present study demonstrated that 19% patients with a single respiratory isolation of MK would progress to MK-PD, and they should be followed for at least 9 months to determine its clinical relevance. Given the high probability of adverse drug reactions, regular monitoring is recommended, rather than immediate anti-MK treatment.

Among patients with a single MK respiratory isolation who developed MK-PD within 1 year, it is striking that all experienced radiographic progression and 44% of them died. The mortality rate in this study is much higher than that in other reports, ranging from 9% to 15.8%^3,23^, probably because the present study was conducted in medical centres where patients tended to have high disease severity. In addition, the diagnosis of MK-PD in the present study strictly followed the American Thoracic Society/Infectious Diseases Society of America guidelines^1^, whereas previous reports exclusively employed the microbiological component of the current guidelines, which may underestimate the severity of disease^9^. Given the poor outcome, predicting subsequent progression to MK-PD in patients with single MK isolation in sputum is crucial for early and accurate initiation of anti-MK treatment.

Different NTM species can coexist in 8% to 25% of patients with NTM infection^16,19^. This highlights the dynamic nature of NTM and puzzles clinicians. However, the clinical significance of this phenomenon is unclear. In patients receiving treatment for MAC-PD, single respiratory isolation of *M. abscessus* probably requires no therapy^17^. However, the change from MAC to *M. abscessus* is usually accompanied by symptomatic and radiographic worsening^24^. Unlike other studies, the present analysis revealed that coexistence of MK and another NTM may be associated with a lower risk of MK-PD and initial high-grade sputum AFS is a risk factor for MK-PD, supporting the hypothesis that quantitative organism load might determine the clinical relevance of NTM-PD^8,11,25^. Coexistence with other NTM species may therefore reflect that MK is not the dominant microorganism.

The epidemiology of MK is predominantly urban and has been associated with high-density and low-income communities^5,7^. The major reservoir has been postulated to include water systems associated with habitation or industry, and infection probably occurs via an aerosol route^1,11,20^. These factors may explain why the incidence of MK-PD was higher among the patients employed in elementary occupations in the present study; such workers extensively use aerosolized water for dust control^5,20,26^.

There are several limitations of the present study. First, the lack of subtyping for MK precluded us from distinguishing its pathogenic (such as MK subtype 1) and nonpathogenic subtypes^27^. Second, we excluded patients providing less than three sputum samples even if single MK isolation was noted, which may have resulted in underestimation of the incidence of subsequent MK-PD. Third, no standardized microbiological or radiographic follow-up protocols were used in this retrospective study. Fourth, the data were retrieved from medical centres in Taiwan and may not be generalizable to all populations.

In conclusion, approximately one-fifth of the patients with single MK isolation from multiple sputum samples progress to MK-PD within 1 year; the majority of progressions occur in the second and third quarters thereafter. However, once MK-PD develops, radiographic progression is inevitable, with 1-year mortality of >40%. Early treatment for MK should be considered for vulnerable populations. Having an elementary occupation, high-grade sputum AFS positivity, and high initial radiographic score (>6 points) are risk factors for MK-PD, whereas coexistence with NTM other than MK is protective.

## Methods

### Study population

This retrospective study was conducted in two medical centres, the National Taiwan University Hospital (NTUH) and Kaohsiung Medical University Hospital (KMUH), and their four branch hospitals. This multicentre study was approved by the medical centres’ institutional review boards (NTUH REC 201508017RIND and KMUH IRB-SV[I]-2015200266) and the need for informed consent was waived because data utilized in this retrospective study have been de-identified.

From January 2008 to December 2016, respiratory specimens were retrieved from the mycobacteriology databases. Mycobacteriologic examinations were performed as described previously^9^. Only patients with new episodes of MK since 2008 were selected^9^. Patients who provided ≥3 sputum samples within 3 months with only one MK isolation, and who had ≥2 follow-up sputum samples and chest radiography in the subsequent 9 months were selected into the study. Only the first episode of single MK isolation for each patient was selected for analysis. We excluded patients lacking demographic data and having TB concomitantly. The index date was defined as the date when the index culture of MK isolation was plated. The patients were followed up until diagnosis of MK-PD, death, or 1 year after the index date.

### Data collection

Patient characteristics, including age, sex, body-mass index (BMI), smoking status, education level, occupation, comorbidities (pulmonary and systemic), symptoms, laboratory data, serial radiographic and microbiological reports, treatment course, and outcome were recorded. The categorical classification of occupation was based on the International Standard Classification of Occupation, 2008^28^, in which elementary occupations included (1) sales and services elementary occupations; (2) agricultural, fishery and related labourers; and (3) labourers in mining, construction, manufacturing and transport.

Chest radiographs and computed tomographic scans were interpreted independently by two pulmonologists. We categorized the patterns as fibrocavitary (FC) and nodular bronchiectatic (NB) and the extent as focal and multifocal. Radiographic scores for severity assessment were recorded as previously described^29^.

The MK treatment administered was analysed in two ways: (1) intention-to-treat analysis for patients who had ever received any drugs against MK, regardless of duration; and (2) per-protocol analysis for patients who had received combination chemotherapy against MK for >2 months in the first 3 months after the index date.

### Outcome assessment

The primary outcome was development of MK-PD within 1 year of the index date. The diagnosis of MK-PD was according to current guidelines^1^. The secondary outcome was radiographic progression and mortality within 1 year for those developing MK-PD. MK was considered the cause of death if no pathogens other than MK were identified and radiographic progression of MK-PD was noted.

### Statistical analysis

Continuous variables are presented as mean ± standard deviation or median with interquartile range and were compared using independent samples *t* tests. Categorical variables are expressed as percentages and were compared using the chi-squared test or Fisher’s exact test, as appropriate. The independent factors associated with development of MK-PD were determined using multivariate logistic regression analysis. Time-to-event curves for development of MK-PD were generated and compared using the log-rank test. Statistical significance was set at *p* < 0.05 (two-sided). All statistical analyses were performed using IBM SPSS version 22.0 (IBM, Armonk, NY, USA).

## Data Sharing Statement

All data were deposited in the Information Technology Office of National Taiwan University Hospital and Statistical Analysis Laboratory, Department of Medical Research, Kaohsiung Medical University Hospital. The data were not available for sharing without permission.
